# Supplementary material for: Efficacy, safety, and economic evaluation of Ojeok-san plus Saengmaek-san for gastroesophageal reflux-induced chronic cough: Protocol for a randomized, double-blind, placebo-controlled, parallel, multicenter, investigator-initiated clinical trial
Source: PLoS One. 2025 Jun 25;20(6):e0325786. doi: 10.1371/journal.pone.0325786 (PMC12193977; doi:10.1371/journal.pone.0325786)
Supplement: S1 File — (DOCX) [file pone.0325786.s001.docx]

**Protocol**

**Efficacy and safety of Ojeok-san plus Saengmaek-san for gastroesophageal reflux-induced chronic cough: a randomized, double-blind, placebo-controlled, parallel, multicenter, investigator-initiated clinical trial**

| Protocol No. | KIOM_22_GERC |
| --- | --- |
| Phase of Study | Investigator-initiated clinical trial |
| Version | 1.0 |
| Date | 2023.09.18 |
| Principle Investigator | 1. Beom-Joon Lee/ Kyung Hee University Korean Medicine Hospital  2. Jae-Woo Park/ Gangdong Kyung Hee University Korean Medicine Hospital  3. Yang-Chun Park/ Daejeon University Daejeon Korean Medicine Hospital  4. Jun-Yong Choi/ Pusan National University Korean Medicine Hospital  5. Tae-Yong Park/ Catholic Kwangdong University International St. Mary's Hospital |

The study protocol will be conducted in accordance with the Declaration of Helsinki and Good Clinical Practice guidelines

**목차**

[Document versions 3](#_Toc186160062)

[Abbreviation 4](#_Toc186160063)

[SYNOPSIS 5](#_Toc186160064)

[Schedule of enrolment, interventions, and assessments 9](#_Toc186160065)

[1. Title of the clinical investigation 11](#_Toc186160066)

[2. Principal investigator, Investigational Site, Sponsor 11](#_Toc186160067)

[2.1. Principal investigator 11](#_Toc186160068)

[2.2. Investigational Sites 11](#_Toc186160069)

[2.3. Sponsor 11](#_Toc186160070)

[3. Background 11](#_Toc186160071)

[4. Objectives 12](#_Toc186160072)

[5. Participants 13](#_Toc186160073)

[5.1. Inclusion Criteria 13](#_Toc186160074)

[5.2. Exclusion Criteria 13](#_Toc186160075)

[5.3. Sample size 14](#_Toc186160076)

[5.4. Recruitment 14](#_Toc186160077)

[6. Trial Design 15](#_Toc186160078)

[6.1. Procedure 15](#_Toc186160079)

[6.2. Randomization and Blinding 15](#_Toc186160080)

[7. Dropout and Early termination criteria 16](#_Toc186160081)

[8. Investigational Product 16](#_Toc186160082)

[8.1. OJS plus SMS 17](#_Toc186160083)

[8.2. Placebo 17](#_Toc186160084)

[9. Procedure 17](#_Toc186160085)

[9.1. Timetable 17](#_Toc186160086)

[9.2. Efficacy Outcome Measures 19](#_Toc186160087)

[9.2.1. Primary Efficacy Outcome 19](#_Toc186160088)

[9.2.2. Secondary efficacy outcome 19](#_Toc186160089)

[9.3. Safety Outcomes 21](#_Toc186160090)

[9.4. Economic evaluation 21](#_Toc186160091)

[10. Statistical analysis 21](#_Toc186160092)

[11. Data collection, management, and monitoring 22](#_Toc186160093)

[12. Ethics approval and dissemination 22](#_Toc186160094)

[13. 참고문헌 24](#_Toc186160095)

# Document versions

| **No** | **Version No.** | **Version Date** | **변경내용** |
| --- | --- | --- | --- |
| 1 | 1.0 | 2023.09.18 | First Version |
|  |  |  |  |

# Abbreviation

| **Abbreviation** | **Definition** |
| --- | --- |
| ALP | Alkaline Phosphatase |
| ALT | Alanine Aminotransferase |
| ANOVA | Analysis of Variance |
| AST | Aspartate Aminotransferase |
| BUN | Blood Urea Nitrogen |
| CBC | Complete Blood Count |
| CRF | Case Report Form |
| CSS | Cough Symptom Score |
| FAS | Full Analysis Set |
| GERDQ | Gastroesophageal reflux disease questionnaire |
| GGT | Gamma-glutamyl transpeptidase |
| HARQ | Hull Airway Reflux (hypersensitivity) Questionnaire |
| HCG | Human Chorionic Gonadotropin |
| ITT | Intention-to-treat |
| LCQ-K | Leicester Cough Questionnaire - Korean |
| NCS | Not clinically significant |
| PP | Per Protocol |
| RBC | Red Blood Cell |
| T-bilirubin | Total Bilirubin |
| U/A | Urinalysis |
| WBC | White Blood Cell |

# SYNOPSIS

| Title of the clinical inves-tigation | Efficacy and safety of Ojeok-san plus Saengmaek-san for gastroesophageal reflux-induced chronic cough: a randomized, double-blind, placebo-controlled, parallel, multicenter, investigator-initiated clinical trial |
| --- | --- |
| Objective | This study is a randomized, double-blind, placebo-controlled, parallel, multi-center, investigator-initiated clinical trial to assess the efficacy, safety, and economic value of OJS plus SMS compared to placebo in patients with GERC. |
| Princi-pal investigator /Investigational Site | 1. Beom-Joon Lee/ Kyung Hee University Korean Medicine Hospital  2. Jae-Woo Park/ Gangdong Kyung Hee University Korean Medicine Hospital  3. Yang-Chun Park/ Daejeon University Daejeon Korean Medicine Hospital  4. Jun-Yong Choi/ Pusan National University Korean Medicine Hospital  5. Tae-Yong Park/ Catholic Kwangdong University International St. Mary's Hospital |
| Participants | Gastroesophageal reflux-induced chronic cough |
| Investigational Product | 1. OJS plus SMS   \| ***Ojeok-san*** \|  \| \| --- \| --- \| \| Atractylodis Rhizoma \| 0.95 \| \| Ephedrae Herba \| 0.2 \| \| Citri Unshius Pericarpium \| 0.4 \| \| Magnoliae Cortex \| 0.08 \| \| Platycodonis Radix \| 0.43 \| \| Aurantii Immaturus Fructus \| 0.31 \| \| Angelicae Gigantis Radix \| 0.37 \| \| Zingiberis Rhizoma \| 0.22 \| \| Paeoniae Radix \| 0.27 \| \| Poria Sclerotium \| 0.02 \| \| Cnidii Rhizoma \| 0.3 \| \| Angelicae Dahuricae Radix \| 0.31 \| \| Pinelliae Tuber \| 0.22 \| \| Cinnamomi Cortex \| 0.04 \| \| Glycyrrhizae Radix et Rhizoma \| 0.2 \| \| Zingiberis Rhizoma Recens \| 0.03 \| \| Total \| 4.35 \| \| ***Saengmaek-san*** \|  \| \| Liriopis Tuber \| 0.75 \| \| Ginseng Radix \| 0.30 \| \| Schisandrae Fructus \| 0.36 \| \| Total \| 1.41 \|   2. Placebo  Placebos do not contain any of the active ingredients, OJS or SMS, and are composed of starch, lactose, and coloring and flavoring agents. |
| Methods | 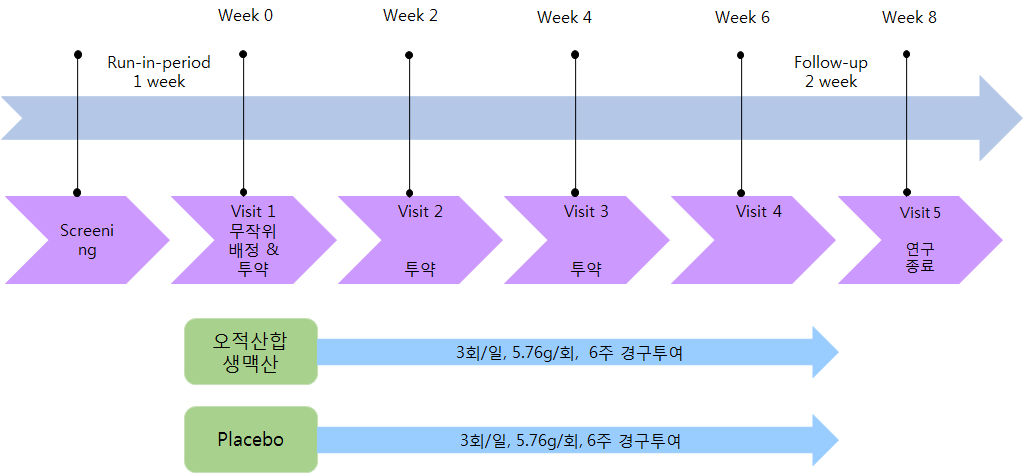  This study is a randomized, double-blind, placebo-controlled, parallel, multi-center, investigator-initiated clinical trial to assess the efficacy, safety, and economic value of OJS plus SMS compared to placebo in patients with GERC. The trial will be conducted at five hospitals in South Korea: Kyung Hee University Korean Medicine Hospital, Gangdong Kyung Hee University Korean Medicine Hospital, Daejeon University Daejeon Korean Medicine Hospital, Pusan National University Korean Medicine Hospital, and Catholic Kwangdong University International St. Mary's Hospital. After participants voluntarily signed a written informed consent, they will be screened for eligibility assessment, and those who meet the inclusion criteria will be enrolled for the next 8 weeks of the trial period (Fig.1). A total of 138 participants will be enrolled in this study from January, 2024. Participants will be allocated to either the OJS plus SMS group or the placebo group in a 1:1 ratio and will be asked to administer the investigational medicine three times a day for 6 weeks. For the evaluation of efficacy, safety, and economic assessments, participants will visit every 2 weeks until the last follow-up visit on week 8. |
| Sample Size | \| OJS plus SMS group \| Placebo group \| Total \| \| --- \| --- \| --- \| \| 69 \| 69 \| 138 \| |
| Inclusion criteria | 1. Aged 19-65 years 2. Subjects who have had a history of cough continuously for > 8 weeks 3. Subjects who had been diagnosed with GERD within the last 1 year (those who submitted documents diagnosed with reflux esophagitis at other hospitals or prescribed reflux esophagitis medicines for more than 4 weeks) 4. Subjects who consent to participate |
| Exclusion criteria | 1. Present with abnormal findings on chest radiography, pulmonary function test, or nasal endoscopy that might lead to cough. 2. Diagnosis of acute respiratory diseases (including upper respiratory tract disorders) within the previous month. 3. Presence of postnasal drip syndrome 4. Diagnosis of chronic respiratory diseases (e.g., chronic obstructive pulmonary disease, bronchial asthma, bronchiectasis, interstitial lung disease, and other chronic respiratory diseases) within the last 2 years 5. Diagnosed with Los Angeles classification system grade C or higher GERD within the past year 6. Symptoms indicative of malignant disease within the GI tract (e.g., severe dysphagia, bleeding, weight loss, anemia, and bloody stools) 7. History of esophagostenosis, esophageal varices, Barrett’s esophagus, peptic ulcer or gastrointestinal bleeding, and Zollinger-Ellison syndrome. 8. History of surgical or endoscopic anti-reflux treatment 9. Treatment with angiotensin-converting enzyme inhibitor during the previous 4 months 10. Treatment with cough medicines, glucocorticoids, leukotriene receptor antagonists, anticholinergic drugs, long-acting β2-agonists within the previous 2 weeks 11. Treatment with antihistamines within the previous 2 weeks 12. Treatment with PPIs, histamine receptor antagonists, mucosa-protective agents, GI motility promoters, antacids, antidepressants, anxiolytics, and lower esophageal sphincter agonists within the previous 2 weeks 13. Treatment with digitalis, mineralocorticoids, anticoagulants, and high-dose aspirin (≤325 mg is allowed) within the previous 4 weeks 14. Treatment with potassium-containing drugs, licorice-containing drugs, glycyrrhizinic acid or its salt-containing drugs, loop diuretics (furosemide, ethacrynic acid), or thiazide diuretics (trichloromethiazide) within the previous four weeks 15. A lifetime smoking history of ≥20 packs (400 cigarettes) 16. Body mass index < 18.5 kg/m^2^ 17. Aspartate aminotransferase (AST) or alanine aminotransferase (ALT) level at least twofold higher than the upper limit of normal or an eGFR ≤ 60 mL/min/1.73㎡ 18. Unregulated hypertension (high blood pressure of 160 mmHg in the condenser or high blood pressure exceeding 100 mmHg in a relaxation period > 3 min) 19. Active infection requiring systemic antibiotic therapy 20. Blood-clotting disorder 21. Hepatitis B (active) or hepatitis C, chronic liver disease 22. History of cerebrovascular accident (CVA) 23. History of malignant tumors (e.g., lung or esophageal cancer) within the last five years 24. History of drug or alcohol abuse 25. Allergies or sensitivities to the experimental medicine/placebo 26. Pregnant or breastfeeding 27. Subjects who did not agree to use contraception by medically permitted methods 28. Subjects who have participated in clinical trials within the past month 29. Subjects who are judged by the investigators to be inappropriate for the clinical trial |
| Efficacy Outcome Measures | - **Primary efficacy outcome**   - Cough Diary Score (CDS)   - **Secondary efficacy outcome**   1. Cough VAS  2. Leicester Cough Questionnaire – Korean version (LCQ-K)  3. Gastroesophageal reflux disease questionnaire (GERDQ)  4. Hull Airway Reflux Questionnaire (HARQ)  5. 5-level EuroQol 5-dimensional questionnaire (EQ-5D-5L)  6. Pattern Identification for Chronic Cough Questionnaire (PICCQ)  7. Pattern Identification for GERD |
| Safety Outcome Measures | - Adverse Events - Vital Signs - Laboratory tests - EKG |

# Schedule of enrolment, interventions, and assessments

|  | **STUDY PERIOD** | | | | | |
| --- | --- | --- | --- | --- | --- | --- |
|  | **Enrolment** | **Allocation** | **Post-allocation** | | | **Follow-up** |
| **VISIT** | ***1*** | **2** | ***3*** | ***4*** | ***5*** | ***6*** |
| **TIMEPOINT** | ***-2~1 week*** | **0** | ***2 weeks ± 3days*** | ***4 weeks ± 3days*** | ***6 weeks ± 3days*** | ***8 weeks ± 3days*** |
| **ENROLMENT:** |  |  |  |  |  |  |
| Eligibility screen | X |  |  |  |  |  |
| Informed consent | X |  |  |  |  |  |
| Demographics | X |  |  |  |  |  |
| Medical and  treatment history | X |  |  |  |  |  |
| Physical examination | X |  |  |  |  |  |
| Vital sign | X | X | X | X | X | X |
| Chest X-ray | X | X | X | X | X | X |
| Pulmonary Function Test | X |  |  |  |  |  |
| FeNO | X |  |  |  |  |  |
| Nasal Endoscopy | X |  |  |  |  |  |
| PNS x-ray (if needed) | X |  |  |  |  |  |
| EKG | X |  |  |  |  |  |
| Laboratory tests | X |  |  |  | X |  |
| Pregnancy Test | X |  |  |  |  |  |
| Allocation |  | X |  |  |  |  |
| **INTERVENTIONS:** |  |  |  |  |  |  |
| OJS plus SMS |  |  |  |  |  |  |
| Placebo |  |  |  |  |  |  |
| **ASSESSMENTS:** |  |  |  |  |  |  |
| Cough Symptom Score | X | X | X | X | X | X |
| Cough VAS | X | X | X | X | X | X |
| LCQ-K |  | X | X | X | X | X |
| GERDQ |  | X | X | X | X | X |
| HARQ |  | X | X | X | X | X |
| Pattern Identification for Chronic Cough Questionnaire | X | X |  |  |  |  |
| Pattern Identification for GERD |  | X |  |  |  |  |
| EQ-5D-5L |  | X | X | X | X | X |
| Abdominal Examination |  | X |  |  |  |  |
| WPAI:SHP |  | X | X | X | X | X |
| Adverse events |  | X | X | X | X | X |
| Compliance test |  |  | X | X | X |  |

# **Title of the clinical investigation**

Efficacy and safety of Ojeok-san plus Saengmaek-san for gastroesophageal reflux-induced chronic cough: a randomized, double-blind, placebo-controlled, parallel, multicenter, investigator-initiated clinical trial

(Protocol Number: KIOM_22_GERC)

# Principal investigator, Investigational Site, Sponsor

## Principal investigator

Beom-Joon Lee/ Kyung Hee University Korean Medicine Hospital

Jae-Woo Park/ Gangdong Kyung Hee University Korean Medicine Hospital

Yang-Chun Park/ Daejeon University Daejeon Korean Medicine Hospital

Jun-Yong Choi/ Pusan National University Korean Medicine Hospital

Tae-Yong Park/ Catholic Kwangdong University International St. Mary's Hospital

## Investigational Sites

Kyung Hee University Korean Medicine Hospital

Gangdong Kyung Hee University Korean Medicine Hospital

Daejeon University Daejeon Korean Medicine Hospital

Pusan National University Korean Medicine Hospital

Catholic Kwangdong University International St. Mary's Hospital

## Sponsor

Korea Institute of Oriental Medicine

# Background

Gastroesophageal reflux-induced chronic cough (GERC) is among the most common extraesophageal manifestations of gastroesophageal reflux disease (GERD), with cough being observed in 30.5–34.9% of patients with GERD ^1^. GERD is also a major cause of chronic cough, along with the upper airway cough syndrome and cough-variant asthma ^2^. GERD and coughing affect each other, aggravating GERC. Under the current understandings, the mechanism by which gastroesophageal reflux induces cough is explained by reflux theory (micro-aspiration of the gastric refluxate into the respiratory tract), reflex theory (the stimulation of the esophageal-bronchial cough reflex mediated by the afferent nerves in the distal esophagus) ^3^, and esophageal dysmotility ^4^. However, a causal relationship between GERD and coughing has not yet been established.

Anti-reflux treatments of GERC, such as proton pump inhibitors (PPIs) and H2 antagonists, alone or in combination with promotility agents, are currently considered the standard therapy for GERC ^3^. However, recent studies have reported that more than one-third of patients do not respond to PPIs ^5^, and multiple reviews have found a lack of evidence regarding the use of empirical PPI therapy for GERC ^6^. These refractory cases of GERC are often observed in patients who do not have GERD symptoms such as heartburn and regurgitation, and are presumed to be due to non-acidic or weakly acidic reflux (4), which accounts for 80% of PPI-treated chronic cough cases ^7^. Recently, for refractory GERC, neuromodulators such as gabapentin and baclofen have been used in some studies; however, their efficacy remains unclear with regard to unnecessary adverse effects ^8^. Therefore, further studies are needed to develop a new, effective, and safe drug to manage GERC, including non-symptomatic refractory GERC.

In this study, we propose the herbal medicines Ojeok-san (OJS) plus Saengmaek-san (SMS) for the treatment of GERC as a new insight. Contrary to anti-reflux drugs, which have limitations in non-symptomatic GERC, we targeted the management of the cough reflex by modulating neurogenic airway inflammation and improving esophageal dysmotility. OJS and SMS are widely used in Korean Medicine to manage digestive, respiratory, and neurological disorders owing to their pharmacological activities ^9 10^. OJS exerts anti-inflammatory effects by decreasing the levels of pro-inflammatory cytokines ^11^ and analgesic effects by ameliorating visceral and somatic nociception ^12^. It has also been reported to reduce airway inflammation and pulmonary fibrosis, by suppressing the level of T helper type 2 cytokines, VEGF, and TGF-β1/Smad3 expressions ^13^. SMS, primarily used for dry cough, is known for its mucolytic effects on the respiratory tract ^14^, antioxidant and anti-inflammatory effects ^10^, and gastroprokinetic effects by regulating gastrointestinal (GI) motility and increasing the activity of the interstitial cells of Cajal ^15^. Taken together, we expect that OJS plus SMS can attenuate airway inflammation and regulate esophageal motility, thereby exerting its effects on GERC.

We found the potential effects of OJS plus SMS for GERC in our pilot study by showing significant differences in cough diary scores (CDS) compared to the placebo ^16^. By addressing the limitations of a previous pilot study, we designed a confirmatory, large-scale, multi-center clinical trial to evaluate the efficacy, safety, and economic value of OJS plus SMS in the treatment of GERC patients. We specified the diagnosis of GERD and the restricted conditions that could affect its safety and efficacy in patients with GERC. Secondary efficacy outcomes evaluating GERD-related symptoms, quality of life, and economic status were added to this trial. Overall, cough symptoms, airway hypersensitivity, GERD-related symptoms, and quality of life will be evaluated in this trial, and our study results will provide evidence for the use of OJS plus SMS in the treatment of GERC.

# Objectives

This study is a randomized, double-blind, placebo-controlled, parallel, multi-center, investigator-initiated clinical trial to assess the efficacy, safety, and economic value of OJS plus SMS compared to placebo in patients with GERC.

# Participants

## Inclusion Criteria

1. Aged 19-65 years
2. Subjects who have had a history of cough continuously for > 8 weeks
3. Subjects who had been diagnosed with GERD within the last 1 year (those who submitted documents diagnosed with reflux esophagitis at other hospitals or prescribed reflux esophagitis medicines for more than 4 weeks)
4. Subjects who consent to participate

## Exclusion Criteria

1. Present with abnormal findings on chest radiography, pulmonary function test, or nasal endoscopy that might lead to cough.
2. Diagnosis of acute respiratory diseases (including upper respiratory tract disorders) within the previous month.
3. Presence of postnasal drip syndrome
4. Diagnosis of chronic respiratory diseases (e.g., chronic obstructive pulmonary disease, bronchial asthma, bronchiectasis, interstitial lung disease, and other chronic respiratory diseases) within the last 2 years
5. Diagnosed with Los Angeles classification system grade C or higher GERD within the past year
6. Symptoms indicative of malignant disease within the GI tract (e.g., severe dysphagia, bleeding, weight loss, anemia, and bloody stools)
7. History of esophagostenosis, esophageal varices, Barrett’s esophagus, peptic ulcer or gastrointestinal bleeding, and Zollinger-Ellison syndrome.
8. History of surgical or endoscopic anti-reflux treatment
9. Treatment with angiotensin-converting enzyme inhibitor during the previous 4 months
10. Treatment with cough medicines, glucocorticoids, leukotriene receptor antagonists, anticholinergic drugs, long-acting β2-agonists within the previous 2 weeks
11. Treatment with antihistamines within the previous 2 weeks
12. Treatment with PPIs, histamine receptor antagonists, mucosa-protective agents, GI motility promoters, antacids, antidepressants, anxiolytics, and lower esophageal sphincter agonists within the previous 2 weeks
13. Treatment with digitalis, mineralocorticoids, anticoagulants, and high-dose aspirin (≤325 mg is allowed) within the previous 4 weeks
14. Treatment with potassium-containing drugs, licorice-containing drugs, glycyrrhizinic acid or its salt-containing drugs, loop diuretics (furosemide, ethacrynic acid), or thiazide diuretics (trichloromethiazide) within the previous four weeks
15. A lifetime smoking history of ≥20 packs (400 cigarettes)
16. Body mass index < 18.5 kg/m^2^
17. Aspartate aminotransferase (AST) or alanine aminotransferase (ALT) level at least twofold higher than the upper limit of normal or an eGFR ≤ 60 mL/min/1.73㎡
18. Unregulated hypertension (high blood pressure of 160 mmHg in the condenser or high blood pressure exceeding 100 mmHg in a relaxation period > 3 min)
19. Active infection requiring systemic antibiotic therapy
20. Blood-clotting disorder
21. Hepatitis B (active) or hepatitis C, chronic liver disease
22. History of cerebrovascular accident (CVA)
23. History of malignant tumors (e.g., lung or esophageal cancer) within the last five years
24. History of drug or alcohol abuse
25. Allergies or sensitivities to the experimental medicine/placebo
26. Pregnant or breastfeeding
27. Subjects who did not agree to use contraception by medically permitted methods
28. Subjects who have participated in clinical trials within the past month
29. Subjects who are judged by the investigators to be inappropriate for the clinical trial

## Sample size

1. **Sample Size**

| OJS plus SMS group | Placebo group | Total |
| --- | --- | --- |
| 69 | 69 | 138 |

1. **Rationale**

Based on our previous study results, we calculated a sample size of 138 (69 participants for each group) for this trial (16). The calculation was conducted to test the difference in the change in CDS from baseline (week 0) to post-dosing of OJS plus SMS (week 6) compared with the placebo. In our preliminary clinical trial, the mean difference between the two groups was found to be 0.75 and the standard deviation was 1.4. We assumed the power to detect the difference to be 0.8, the two-sided significance level to be 0.05, and a dropout rate of 20% to determine the sample size.

## Recruitment

Participants will be recruited from the outpatient departments of Pulmonology or Gastroenterology at each affiliated hospital. Recruitment brochures will be posted on the bulletin boards and websites of each hospital, and advertisements in mass media or local communications will be conducted as needed to promote recruitment.

# Trial Design

## Procedure

This study is a randomized, double-blind, placebo-controlled, parallel, multi-center, investigator-initiated clinical trial to assess the efficacy, safety, and economic value of OJS plus SMS compared to placebo in patients with GERC. The trial will be conducted at five hospitals in South Korea: Kyung Hee University Korean Medicine Hospital, Gangdong Kyung Hee University Korean Medicine Hospital, Daejeon University Daejeon Korean Medicine Hospital, Pusan National University Korean Medicine Hospital, and Catholic Kwangdong University International St. Mary's Hospital. After participants voluntarily signed a written informed consent, they will be screened for eligibility assessment, and those who meet the inclusion criteria will be enrolled for the next 8 weeks of the trial period (Fig.1). A total of 138 participants will be enrolled in this study from January, 2024. Participants will be allocated to either the OJS plus SMS group or the placebo group in a 1:1 ratio and will be asked to administer the investigational medicine three times a day for 6 weeks. For the evaluation of efficacy, safety, and economic assessments, participants will visit every 2 weeks until the last follow-up visit on week 8.


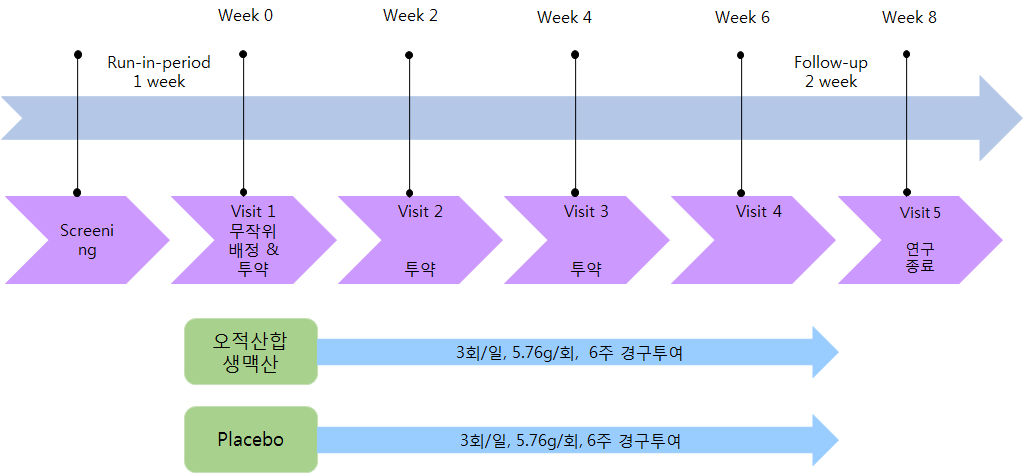


<임상시험 진행 흐름>

## Randomization and Blinding

An independent statistician conducted randomization using a computer random number generator of SAS® Version 9.4 (SAS institute. Inc., Cary, NC, USA) using the block randomization method. The allocation ratio was 1:1 for each intervention (OJS plus SMS) and control (placebo) group. The generated random sequence was transmitted to the manufacturer responsible for labeling the participants’ identification codes on each investigational medicine package. These labelled medicines will be delivered to management pharmacists at each hospital, and pharmacists will give the corresponding investigational medicine to each participant using their identification code. The random number will be kept by an independent investigator who has no relationship with the patient throughout the trial and will only be disclosed when a serious adverse event occurs.

All investigators, participants, and outcome assessors will be blinded. The intervention and placebo drugs are distinguished only by the participants’ identification codes, labelled in identical and opaque packages, and both drugs were manufactured with the same color, taste, and smell.

# Dropout and Early termination criteria

At any time during the clinical trial, participants can voluntarily withdraw from the clinical trial. Additionally, anyone who meets any of the following conditions will be dropped out from the trial at the discretion of investigators:

1. found to violate the inclusion and exclusion criteria after screening visit,
2. in the case where the participant requests discontinuation of the trial or refuses the treatment due to unsatisfactory treatment effects during the trial period,
3. in the case where the participant cannot be tracked during the trial period,
4. when it is judged by investigators that trial participation is no longer possible due to an adverse reaction or a concomitant disease,
5. in the case of taking prohibited drugs that may affect the trial result without the instruction of investigators during the trial period,
6. when it is judged that the effect evaluation of the clinical trial is not appropriate because the overall compliance with the investigational products is less than 70% in YGJ and PWS groups,
7. when the participant violates the trial plan, and it is difficult for the participant to continue the trial, and
8. when investigators judged that the progress of the clinical trial is no more appropriate.

In principle, this clinical trial is finalized when the registration of the planned number of participants is completed and the completeness of the collected data is secured. However, in the following cases, this clinical trial can be discontinued: (1) when the harmfulness of the supplied investigational products is found, (2) if moderate or severe adverse events judged to be related to investigational products occur in more than 25% of all participants, and (3) in the case when the targeted participants are not registered even though a sufficient recruitment period has been given.

# Investigational Product

## OJS plus SMS

| ***Ojeok-san*** |  |
| --- | --- |
| Atractylodis Rhizoma | 0.95 |
| Ephedrae Herba | 0.2 |
| Citri Unshius Pericarpium | 0.4 |
| Magnoliae Cortex | 0.08 |
| Platycodonis Radix | 0.43 |
| Aurantii Immaturus Fructus | 0.31 |
| Angelicae Gigantis Radix | 0.37 |
| Zingiberis Rhizoma | 0.22 |
| Paeoniae Radix | 0.27 |
| Poria Sclerotium | 0.02 |
| Cnidii Rhizoma | 0.3 |
| Angelicae Dahuricae Radix | 0.31 |
| Pinelliae Tuber | 0.22 |
| Cinnamomi Cortex | 0.04 |
| Glycyrrhizae Radix et Rhizoma | 0.2 |
| Zingiberis Rhizoma Recens | 0.03 |
| Total | 4.35 |
| ***Saengmaek-san*** |  |
| Liriopis Tuber | 0.75 |
| Ginseng Radix | 0.30 |
| Schisandrae Fructus | 0.36 |
| Total | 1.41 |

## Placebo

Placebos do not contain any of the active ingredients, OJS or SMS, and are composed of starch, lactose, and coloring and flavoring agents.

# Procedure

## Timetable

|  | **STUDY PERIOD** | | | | | |
| --- | --- | --- | --- | --- | --- | --- |
|  | **Enrolment** | **Allocation** | **Post-allocation** | | | **Follow-up** |
| **VISIT** | ***1*** | **2** | ***3*** | ***4*** | ***5*** | ***6*** |
| **TIMEPOINT** | ***-2~1 week*** | **0** | ***2 weeks ± 3days*** | ***4 weeks ± 3days*** | ***6 weeks ± 3days*** | ***8 weeks ± 3days*** |
| **ENROLMENT:** |  |  |  |  |  |  |
| Eligibility screen | X |  |  |  |  |  |
| Informed consent | X |  |  |  |  |  |
| Demographics | X |  |  |  |  |  |
| Medical and  treatment history | X |  |  |  |  |  |
| Physical examination | X |  |  |  |  |  |
| Vital sign | X | X | X | X | X | X |
| Chest X-ray | X | X | X | X | X | X |
| Pulmonary Function Test | X |  |  |  |  |  |
| FeNO | X |  |  |  |  |  |
| Nasal Endoscopy | X |  |  |  |  |  |
| PNS x-ray (if needed) | X |  |  |  |  |  |
| EKG | X |  |  |  |  |  |
| Laboratory tests | X |  |  |  | X |  |
| Pregnancy Test | X |  |  |  |  |  |
| Allocation |  | X |  |  |  |  |
| **INTERVENTIONS:** |  |  |  |  |  |  |
| OJS plus SMS |  |  |  |  |  |  |
| Placebo |  |  |  |  |  |  |
| **ASSESSMENTS:** |  |  |  |  |  |  |
| Cough Symptom Score | X | X | X | X | X | X |
| Cough VAS | X | X | X | X | X | X |
| LCQ-K |  | X | X | X | X | X |
| GERDQ |  | X | X | X | X | X |
| HARQ |  | X | X | X | X | X |
| Pattern Identification for Chronic Cough Questionnaire | X | X |  |  |  |  |
| Pattern Identification for GERD |  | X |  |  |  |  |
| EQ-5D-5L |  | X | X | X | X | X |
| Abdominal Examination |  | X |  |  |  |  |
| WPAI:SHP |  | X | X | X | X | X |
| Adverse events |  | X | X | X | X | X |
| Compliance test |  |  | X | X | X |  |

## Efficacy Outcome Measures

### Primary Efficacy Outcome

#### Cough symptom score (Cough diary)

The CDS is a subjective cough score questionnaire that assesses the severity and frequency of cough on five scales (0 to 4) during the daytime and nighttime ^18^. The cough severity scale is divided into 0: none, 1: slight, 2: mild, 3: moderate, and 4: severe, whereas the cough frequency ranges between 0 (none), 1 (infrequent/occasional), 2 (several times), 3 (many times), and 4 (all the time). Each of the daytime and nighttime scores is calculated by adding the score of cough severity and cough frequency, with a maximum score of 8; the total CDS is an averaged score of daytime and nighttime CDS. All participants will be instructed to record their CDS twice a day: daytime score assessing symptoms occurring from 8am to 8pm and nighttime score from 8pm to 8am. At every visit, a Cough Diary will be given to each participant to record their symptoms until the next visit, and the average score between visits will be used as the outcome to evaluate the efficacy of OJS plus SMS compared with the placebo.

The primary outcome of this trial is the change in CDS between baseline (pre-drug) and week 6 (post-drug) in the OJS plus SMS group compared to the placebo group. CDS was chosen as the primary outcome measure, as a subjective cough score is the most frequently used outcome in clinical trials related to cough ^19^ and was also the primary outcome in our pilot trial showing significant differences between OJS plus SMS and placebo.

### Secondary efficacy outcome

#### Cough VAS (Cough diary)

The cough VAS is another cough score outcome widely used to assess acute or chronic cough ^20 21^. It is also highly responsible for changes in cough severity, making it easy to compare therapeutic effects in clinical trials ^22^. The participants were asked to mark the severity of cough on the line with a calibration of 0 to 100 mm, with 0 indicating ‘no cough’ and 10 indicating ‘unbearable cough.’ The distance between zero and the marked point will be measured using the cough visual analog scale score. Participants will be instructed to record the cough VAS daily in the given cough diary for the next 2 weeks, and the average score will be used as the outcome.

#### Leicester Cough Questionnaire-Korean (LCQ-K)

The LCQ-K, which consists of 19 items, is a quality of life (QoL) questionnaire specified for cough symptoms. Each item ranges from 1 to 7, with higher scores indicating a better quality of life and is divided into three domains: physical, mental, and social ^23^. Previous studies have demonstrated a relationship between the LCQ score and the severity of cough, and it is also recommended by the European Respiratory Society as a QoL outcome for the impact of cough in patients with chronic cough ^24^. The validated Korean version of the LCQ will be used in the trials at each visit ^25^.

#### Gastroesophageal reflux disease questionnaire (GERDQ)

The GERDQ is a validated patient-report questionnaire for diagnosis and management of GERD ^26^. It includes six items related to symptoms such as heartburn, regurgitation, upper stomach pain, nausea, night sleep disturbance, and the need for additional medication. Each item is graded from 0 to 3 according to the frequency of symptoms in the past 1 week, with a higher score indicating more severe GERD-related symptoms. As the GERDQ is not only used for assessing changes in treatment outcomes but also for diagnosing GERD, it will be useful to identify GERD severity in patients enrolled in our trial as none, mild, moderate, or severe ^27^. Therefore, it can be used to compare the efficacy of OJS plus SMS in symptomatic and non-symptomatic patients with GERD.

#### Hull Airway Reflux (hypersensitivity) Questionnaire (HARQ)

The HARQ is designed to measure cough hypersensitivity and contains 14 items graded from 0 to 5, with a maximum score of 70 points. Higher scores indicated higher sensitivity of the airway ^28^. As cough hypersensitivity is highly related to chronic cough and represents a distinct clinical entity, the HARQ was used to assess patients with GERC in this trial.

#### Pattern Identification for Chronic Cough Questionnaire (PICCQ)

The Pattern Identification for Chronic Cough Questionnaire will be assessed at baseline to compare the differences in efficacy by pattern identification. Based on the pattern identification for chronic cough, participants will be classified into one of five patterns: cold wind, phlegm turbidity, fire heat, lung deficiency, or kidney yang deficiency ^30^. These pattern identifications are the diagnostic and treatment criteria used in Korean Medicine to determine appropriate therapies for each patient. Thus, it is important to assess the relationship between these patterns and the therapeutic efficacy of OJS plus SMS.

#### Pattern Identification for GERD

Pattern identification for GERD will also be performed in all participants at baseline. We will investigate the distribution of pattern identification in patients with GERC and the pattern identification that best responds to OJS plus SMS. Pattern identification of GERD includes stagnation of Liver Qi, stomach yin deficiency, spleen–stomach weakness, and spleen-stomach dampness-heat. The questionnaire contains 40 items, of which 23 are self-report items and 8 are assessed by a Korean Medicine Doctor ^31^.

## Safety Outcomes

1. Adverse Events
2. vital signs (blood pressure, body temperature, pulse rate, and respiratory rate)
3. EKG
4. Laboratory tests

For the safety outcomes, vital signs (blood pressure, body temperature, pulse rate, and respiratory rate), adverse events, and laboratory test will be evaluated during the trial period. At every visit, vital signs and adverse events will be assessed, and laboratory tests will be conducted before and after drug administration. Liver function tests (aspartate aminotransferase, alanine aminotransferase, alkaline phosphatase, gamma-glutamyl transferase, bilirubin, and protein/albumin), renal function tests (blood urea nitrogen and creatinine), electrolyte content (sodium, potassium, and chloride), blood coagulation tests (prothrombin time and activated partial thromboplastin time), and complete blood count will be performed. Adverse events will be collected by asking participants whether any unfavorable and unintended signs, symptoms, or diseases presented after taking the investigational medicine. These will then be assessed for severity and causality of investigational drugs, and investigators will follow up until the adverse events end.

## Economic evaluation

We will conduct an economic evaluation from the healthcare system and societal perspective over 8 weeks of the trial period in parallel with a randomized controlled trial (RCT). We will estimate cost-utility as the primary economic endpoint and cost-effectiveness as a secondary economic endpoint of OJS plus SMS compared with placebo. Utility data will be collected from trial outcomes of the EQ-5D-5L, a health-related quality of life questionnaire assessed at every visit, and quality-adjusted life years (QALYs) will be calculated to estimate the incremental cost-utility ratio. Effectiveness data will also be obtained from the efficacy outcomes in the main RCT, including the CDS, cough VAS, LCQ-K, GERDQ, and HARQ scores, which will be used to estimate the incremental cost-effectiveness ratio. Cost data will be obtained in terms of medical and non-medical costs (transportation, time, and nursing care costs) and productivity loss costs from institutional data, a specially developed questionnaire for cost, and data from Statistics Korea, if needed. Additionally, sensitivity analysis will be performed on possible variables using one-way sensitivity analysis and probability sensitivity analysis with representative values and the distribution of variables.

# Statistical analysis

Statistical analysis will be performed by an independent statistician using SAS Analytics Pro software and significance will be accepted at an α-level of 0.05 in a two-sided test. For all data analyses, full analysis (FAS) set will be the primary analysis set in this trial, and a per-protocol (PP) set will be performed as an additional analysis. In this trial, the FAS set included all randomly assigned participants who had been evaluated for efficacy at least once after the administration of the investigational medicine. PP sets were defined as participants who completed the trial without violating the protocol, with compliance lower than 75%, taking prohibited medications, or presenting with serious adverse events. Continuous variables are presented as means and 95% confidence intervals and categorical variables as frequencies and percentages.

For primary efficacy analysis, the changes in CDS between baseline and week 6 of OJS plus SMS compared with placebo will be evaluated using a mixed-effect model repeated measure (MMRM), with each group and visit set as fixed effects and participants as random effects. Secondary efficacy analysis, comparing cough VAS, LCQ-K, GERDQ, HARQ, and EQ-5D-5L evaluated at each visit point with baseline between groups, will also be performed using MMRM. The analysis of the changes in each efficacy outcome within the group will be performed using a paired t-test or Wilcoxon signed-rank test, depending on its normality. For missing values, a multiple imputation will be performed. Additionally, to analyze the trends of CDS over time compared with the baseline, RM-ANOVA will be conducted, and Dunnett’s procedure (based on the baseline) will be used as a multiple comparison correction. A safety analysis will be conducted to compare the number of adverse events between groups using Fisher’s exact test.

# Data collection, management, and monitoring

The principal investigator should manage all clinical trial data in compliance with Good Clinical Practice guidelines and related regulations regarding collection, recording, and reporting. Clinical data collected throughout the trial will be entered into an electronic Case Report Form (eCRF) and managed by a data manager. All source documents must be stored in a locked place and security must be maintained by storing them on a computer with limited access to those not related to the trial. These documents will be preserved for 3 years from the end of the clinical trial.

When recording a participant’s personal information, all data will be handled in accordance with the relevant regulations to ensure confidentiality. All records identifying the personal information of all participants will be managed and evaluated according to the participants’ identification numbers and the initials assigned at the start of the study. Only the monitor, the person conducting the inspection, the review committee, or the Minister of Food and Drug Safety can view medical records and clinical trial data within the scope set by relevant regulations without violating test subject confidentiality.

Data monitoring will be conducted during regular and occasional visits by a clinical research associate (CRA) at the Korea Institute of Oriental Medicine. Compliance with the clinical trial protocol, collection of appropriate and accurate data, inspection of informed consent forms, collection and reporting of (serious) adverse reactions, and management of investigational drugs will be confirmed and inspected according to the monitoring plan.

# Ethics approval and dissemination

The study protocol(Ver. 1.2) was authorized by the Ministry of Food and Drug Safety of Korea (MFDS) (approval number 101726) and approved by the Institutional Review Board of Kyung Hee University Korean Medicine Hospital (KOMCIRB2022-11-006), Gangdong Kyung Hee University Korean Medicine Hospital (KHNMCOH2023-10-004), Daejeon University Daejeon Korean Medicine Hospital (DJDSKH-23-DR-10), Pusan National University Korean Medicine Hospital (PNUKHIRB2023-10-003), and Catholic Kwandong University International St. Mary's Hospital (IS23EIME0065). This study was registered with the Clinical Research Information Service (KCT0008908).

Prior to trial screening, all participants will be provided with all information related to the clinical trial with sufficient time and opportunity to ask questions, decide whether to participate, and will be asked to sign written informed consent. They will also be promptly informed of any changes in the clinical trial-related information, even during the trial period. After the trial ends, we will disseminate the study results through peer-reviewed journals, conference presentations, and clinical research information services.

# References

1. Chang, A. B., Lasserson, T. J., Gaffney, J., Connor, F. L., & Garske, L. A. (2011). Gastro-oesophageal reflux treatment for prolonged non-specific cough in children and adults. Cochrane Database Syst Rev(1), Cd004823. doi:10.1002/14651858.CD004823.pub4
2. Chung, K. F., & Pavord, I. D. (2008). Prevalence, pathogenesis, and causes of chronic cough. The Lancet, 371(9621), 1364-1374. doi:http://dx.doi.org/10.1016/S0140-6736(08)60595-4
3. Irwin, R. S., & Madison, J. M. (2000). The diagnosis and treatment of cough. New England Journal of Medicine, 343(23), 1715-1721.
4. Morice, A., Fontana, G., Belvisi, M., Birring, S., Chung, K., Dicpinigaitis, P., Tatar, M. (2007). ERS guidelines on the assessment of cough. European Respiratory Journal, 29(6), 1256-1276.
5. Morice AH, Faruqi S, Wright CE, Thompson R, Bland JM. (2011) Cough hypersensitivity syndrome: a distinct clinical entity. Lung. 189
6. Palombini, B. C., Villanova, C. A. n. C., Araújo, E., Gastal, O. v. L., Alt, D. C., Stolz, D. P., & Palombini, C. O. (1999). A pathogenic triad in chronic cough*: Asthma, postnasal drip syndrome, and gastroesophageal reflux disease. Chest, 116(2), 279-284. doi:10.1378/chest.116.2.279
7. Pratter, M. R., Brightling, C. E., Boulet, L. P., & Irwin, R. S. (2006). An empiric integrative approach to the management of cough : Accp evidence-based clinical practice guidelines. Chest, 129(1_suppl), 222S-231S. doi:10.1378/chest.129.1_suppl.222S
8. Shaheen, N. J., Crockett, S. D., Bright, S. D., Madanick, R. D., Buckmire, R., Couch, M., . . . Henke, D. (2011). Randomised clinical trial: high‐dose acid suppression for chronic cough – a double‐blind, placebo‐controlled study. Alimentary pharmacology & therapeutics, 33(2), 225-234.
9. Sik Shin, I., Young Lee, M., Young Jeon, W., Choon Kim, J., & Kyoo Shin, H. (2013). Ojeok-san, a traditional Korean herbal medicine attenuates airway inflammation and pulmonary fibrosis induced by repeated ovalbumin challenge. Journal of Ethnopharmacology, 149(1), 281-287.
10. Gong EJ, Jung KW, Min YW, Hong KS, Jung HK, Son HJ, et al. Validation of the Korean Version of the Gastroesophageal Reflux Disease Questionnaire for the Diagnosis of Gastroesophageal Reflux Disease. J Neurogastroenterol Motil. 2019;25(1):91-9.
11. 윤지연, & 윤용갑. (2012). 오적산(五積散)의 임상(臨床) 활용(活用)에 대한 방제학적(方劑學的) 고찰(考察). 대한한의학방제학회지, 20(2), 153-164.
12. 이승덕, 김은정, 정찬영, 신경민, 장민기, 윤은혜, 김갑성. (2010). 오적산의 치료 효과 평가에 활용될 변증 설문지 개발을 위한 타당성 문항 추출 연구. 대한한의학회지, 31(4), 101-114.
13. 李梴, 陳柱杓. 新對譯編註 醫學入門. Seoul: 法仁文化社; 2009, p. 1319
14. 汪昻, 蔡仁植, 孟華燮. (國譯) 醫方集解. Seoul: 大星文化社; 1992, p. 361
15. Ko KM. Shengmai san. New York: Taylor & Francis; 2002, p. 136
16. 杨爱武, 吕宏升, 张江灵, 林志敏, 梁仁佩. 生脉散加味对晚期肺腺癌患者化疗毒副反应及临床症状、生活质量的影响. 现代中西医结合杂志. 2016(33).
17. 王玲, 居来提·赛买提, 杨卫江. 生脉散加味治疗结缔组织病合并肺间质纤维化. 中国现代医生. 2011(11).
18. Kim BJ (2013). Shengmaisan Regulates Pacemaker Potentials in Interstitial ells of Cajal in Mice. Journal of pharmacopuncture. 16(4), 36-42.
19. Lee MC, Park JR, Shim JH, Ahn TS, Kim BJ (2015). Effects of Traditional Chinese Herbal Medicine Shengmai-San and Pyungwi-San on Gastrointestinal Motility in Mice. J Korean Med Obes Res. 15(2).
20. 이준환, 방연희, 김재효, 도하윤, 김관일, 정희재, 이범준. (2017). 오적산합생맥산으로 호전된 위식도 역류에 의한 만성기침 환자 3례. 대한한방내과학회지, 38(4), 520-530.
21. Lyu, Y. R., Kim, K. I., Yang, C., Jung, S. Y., Kwon, O. J., Jung, H. J., ... & Lee, B. J. (2022). Efficacy and Safety of Ojeok-San Plus Saengmaek-San for Gastroesophageal Reflux-Induced Chronic Cough: A Pilot, Randomized, Double-Blind, Placebo-Controlled Trial. Frontiers in pharmacology, 616.
22. 김관일, 신승원, 이나라, 이범준, 정희재, 정승기, 이준희. (2015). 만성기침 변증도구 개발을 위한 기초 연구. 대한한방내과학회지. 36(1), 22-39.
23. 한가진, 임정태, 이나라, 김진성, 박재우, 이준희. (2015). 위식도역류질환 변증도구 개발 연구. 대한한방내과학회지, 36(2), 122-152.
24. Birring, S.S., Prudon, B., Carr, A.J., Singh, S.J., Morgan, M.D., Pavord, I.D., (2003). Development of a symptom specific health status measure for patients with chronic cough: Leicester Cough Questionnaire (LCQ). Thorax 58, 339-343.
25. Ours TM, Kavuru MS, Schilz RJ, Richter JE.(1999) A prospective evaluation of esophageal testing and a double-blind, randomized study of omeprazole in a diagnostic and therapeutic algorithm for chronic cough. Am J Gastroenterol. 94(11), 3131-3138.
26. Julious SA. (2005). Sample size of 12 per group rule of thumb for a pilot study. Pharmaceutical statistics. 4, 287-291.
27. Irwin, R. S., French, C. L., Curley, F. J., Zawacki, J. K., & Bennett, F. M. (1993). Chronic Cough Due to Gastroesophageal Reflux: Clinical, Diagnostic, and Pathogenetic Aspects. Chest, 104(5), 1511-1517. doi:http://dx.doi.org/10.1378/chest.104.5.1511
28. Saritas Yuksel, E., & Vaezi, M. F. (2012). Extraesophageal manifestations of gastroesophageal reflux disease: cough, asthma, laryngitis, chest pain. Swiss Med Wkly, 142, w13544. doi:10.4414/smw.2012.13544
29. Irwin, R. S., Baumann, M. H., Bolser, D. C., Boulet, L.-P., Braman, S. S., Brightling, C. E. Tarlo, S. M. (2006). Diagnosis and management of cough executive summary : Accp evidence-based clinical practice guidelines. Chest, 129(1_suppl), 1S-23S. doi:10.1378/chest.129.1_suppl.1S
30. Laukka, M. A., Cameron, A. J., & Schei, A. J. (1994). Gastroesophageal reflux and chronic cough: which comes first? Journal of clinical gastroenterology, 19(2), 100-104.
31. Baldi, F., Cappiello, R., Cavoli, C., Ghersi, S., Torresan, F., & Roda, E. (2006). Proton pump inhibitor treatment of patients with gastroesophageal reflux-related chronic cough: A comparison between two different daily doses of lansoprazole. World Journal of Gastroenterology : WJG, 12(1), 82-88. doi:10.3748/wjg.v12.i1.82
32. SJ, N., Park SC, & Lee SJ. (2016). Extraesophageal Manifestations of Gastroesophageal Reflux Disease. [Extraesophageal Manifestations of Gastroesophageal Reflux Disease]. Korean Journal of Medicine, 91(3), 257-263.
33. Kim, H., & Sung, M.-W. (2015). Pathophysiology, Causes and Treatment of Chronic Cough in Adults: Literature Review. Korean J Otorhinolaryngol-Head Neck Surg, 58(11), 744-753.
